# The ability of callus tissues induced from three *Allium* plants to accumulate health-beneficial natural products, *S*-alk(en)ylcysteine sulfoxides

**DOI:** 10.1007/s11418-022-01631-4

**Published:** 2022-06-13

**Authors:** Naoko Yoshimoto, Takashi Asano, Ayuna Kisanuki, Chihiro Kanno, Machiko Asanuma, Mami Yamazaki, Isao Fujii, Kazuki Saito

**Affiliations:** 1 Graduate School of Pharmaceutical Sciences, Chiba University, 1-8-1 Inohana, Chuo-ku, Chiba, 260-8675 Japan; 2 Plant Molecular Science Center, Chiba University, 1-8-1 Inohana, Chuo-ku, Chiba, 260-8675 Japan; 3 School of Pharmacy, Iwate Medical University, 1-1-1 Idaidori, Yahaba, Iwate, 028-3694 Japan; 4 RIKEN Center for Sustainable Resource Science, 1-7-22 Suehiro-cho, Tsurumi-ku, Yokohama, 230-0045 Japan

**Keywords:** *Allium cepa*, *Allium fistulosum*, *Allium tuberosum*, Callus, *S*-Alk(en)ylcysteine sulfoxide

## Abstract

*S*-Alk(en)ylcysteine sulfoxides (CSOs), such as methiin, alliin, and isoalliin, are health-beneficial natural products biosynthesized in the genus *Allium*. Here, we report the induction of multiple callus tissue lines from three *Allium* vegetables, onion (*A. cepa*), Welsh onion (*A. fistulosum*), and Chinese chive (*A. tuberosum*), and their ability to accumulate CSOs. Callus tissues were initiated and maintained in the presence of picloram and 2-isopentenyladenine as auxin and cytokinin, respectively. For all plant species tested, the callus tissues almost exclusively accumulated methiin as CSO, while the intact plants contained a substantial amount of isoalliin together with methiin. These results suggest that the cellular developmental conditions and the regulatory mechanisms required for the biosynthesis of methiin are different from those of alliin and isoalliin. The methiin content in the callus tissues of onion and Welsh onion was much higher compared to that in the intact plants, and its cellular concentration could be estimated as 1.9–21.7 mM. The activity of alliinase that degrades CSOs in the callus tissues was much lower than that of the intact plants for onion and Welsh onion, but at similar levels as in the intact plants for Chinese chive. Our findings that the callus tissues of onion and Welsh onion showed high methiin content and low alliinase activity highlighted their potential as a plant-based system for methiin production.

## Introduction

The genus *Allium* in the Amaryllidaceae family is one of the largest plant genera, including flavoring vegetables, such as onion (*Allium cepa*) and garlic (*A. sativum*) [1]. The metabolic feature of *Allium* plants is the synthesis of sulfur-containing secondary metabolites, *S*-alk(en)ylcysteine sulfoxides (CSOs), which cause both distinctive flavor and health-promoting effects. To date, *S*-methylcysteine sulfoxide (methiin), *S*-allylcysteine sulfoxide (alliin), *S*-*trans*-1-propenylcysteine sulfoxide (isoalliin), and *S-n*-propylcysteine sulfoxide (propiin) have been identified as the four major CSOs present in *Allium* plants [2–4].

In intact plant tissues, CSOs are stored in the cytosol, whereas alliinase (EC 4.4.1.4), a C–S lyase that cleaves the C–S bond of CSOs, is sequestered in the vacuole of the vascular bundle sheath cells [5–7]. When tissues are damaged, for example, by natural enemies or cooking, alliinase contacts CSOs to generate unstable sulfenic acids, which are immediately converted into various sulfur-containing compounds with biological activities, such as flavoring, antibacterial, antiviral, antifungal, anticancer, antithrombotic, cholesterol-lowering, and antihypertensive effects [3, 4]. Therefore, CSOs and alliinase are the two key components that determine the value of *Allium* plants as flavoring vegetables and the source of health-promoting natural compounds.

**Fig. 1:**
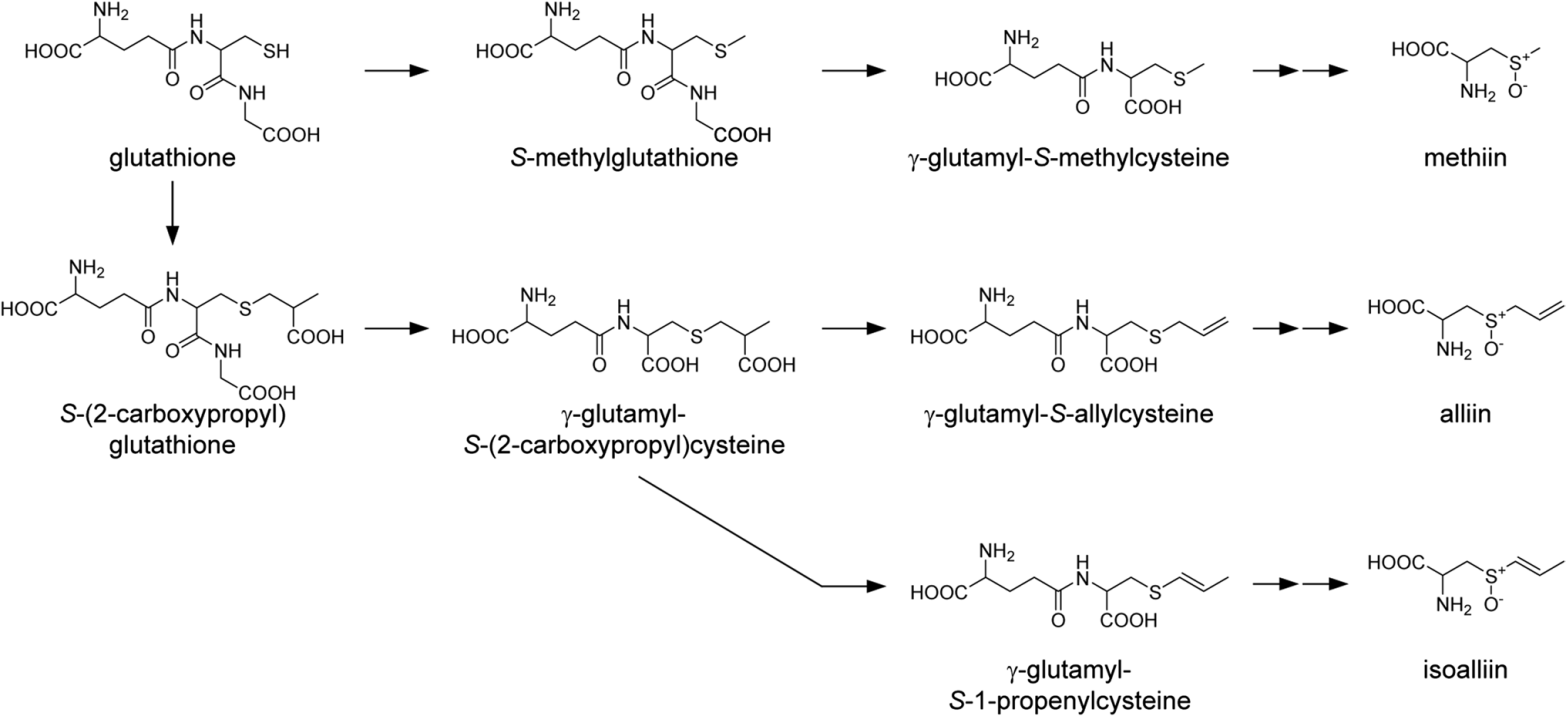
Proposed biosynthetic pathway of methiin, alliin, and isoalliin in *Allium* plants

The biosynthetic pathway of CSOs has been proposed based on previous chemical and radiotracer analyses [8–11]. The proposed pathway is initiated by *S*-alk(en)ylation of the cysteine residue in glutathione, followed by multiple reactions involving modification of the *S*-substituted group, removal of the glycyl and γ-glutamyl groups, and *S*-oxygenation [4, 11]. In this pathway, the methiin biosynthesis separates from the routes for alliin and isoalliin at the initial stage, while the routes for alliin and isoalliin separate at the mid-stage (Fig. 1). In parallel, the pathway through direct *S*-alk(en)ylation of cysteine or thioalk(en)ylation of *O*-acetylserine followed by *S*-oxygenation may be present [10]. Most of these reactions are thought to be catalyzed by specific enzymes. However, only γ-glutamyl transpeptidases and an *S*-oxygenase for alliin biosynthesis in garlic have been identified [12, 13]. Thus, it is difficult at present to generate valuable plants showing modified CSOs levels and microbial systems synthesizing CSOs using transgenic and gene-editing technologies.

Plant cell culture is a promising tool for studying and producing secondary metabolites [14]. Regarding the sulfur-containing secondary metabolites, asparaptine A, a conjugate of arginine and asparagusic acid with inhibitory activity against the angiotensin-converting enzyme, was accumulated in the callus cultures of asparagus (*Asparagus officinalis*) at a rate approximately twice as high as that in edible spears of intact plants [15, 16]. Glucosinolates, which are sulfur-rich amino acid derivatives with cancer-preventive effects, were produced in the cell cultures of Brassicaceae species; however, the levels of glucosinolates were lower in cell cultures than in intact plants [17].

Efforts have been made to establish callus tissues of *Allium* plants, especially onion and garlic, with the aim of evaluating their ability to produce CSOs and alliinase. Intact plants of onion and garlic produce isoalliin and alliin as major CSOs, respectively, and methiin is a minor CSO [1]. In contrast, undifferentiated callus tissues of onion and garlic contain a small amount of methiin and a negligible amount of isoalliin and alliin [18–21]. The content of methiin and alliin was higher in green callus tissues than that in white callus tissues of garlic [19]. Although these observations suggest that the biosynthesis of methiin is carried out even in undifferentiated cells at a low rate, the biosynthesis of isoalliin and alliin requires adequately differentiated cellular environments containing chloroplasts. Differentiation states of cells also seem to be important for the alliinase activity because the alliinase activity of undifferentiated callus tissues of onion was higher than that of dormant onion bulbs, but far lower than that of sprouting bulbs and seedlings of onion [18]. These studies provided basic information on the production of CSOs and alliinase in *Allium* callus tissues; however, the following issues remain to be addressed. First, these studies analyzed only one or a few representative lines of callus tissue. Because the characteristics of callus tissues often vary among lines, analysis of a larger number of callus lines is desirable. Second, some of these studies measured the content of CSOs using TLC, which is semi-quantitative. An analysis using more quantitative methods is preferable. Third, although previous studies have focused on onion and garlic, analysis of other *Allium* species will lead to further understanding of the CSOs and alliinase production in *Allium* plants.

In the present study, we successfully established multiple callus tissue lines from three *Allium* vegetables, onion, Welsh onion (*A. fistulosum*), and Chinese chive (*A. tuberosum*) and analyzed their CSOs levels by liquid chromatography-mass spectrometry (LC–MS) and alliinase activities. We found that the callus tissues efficiently biosynthesized methiin but not alliin and isoalliin. The callus tissues of onion and Welsh onion showed higher methiin levels and lower alliinase activity than the intact plants, demonstrating the potential of these callus tissues as an efficient production system for the natural form of methiin.

## Materials and methods

### Reagents

The natural forms of methiin and isoalliin used as LC–MS standards were kindly provided by House Food Group Inc. (Tokyo, Japan). The natural form of alliin for use as an LC–MS standard was kindly provided by Wakunaga Pharmaceutical Co., Ltd. (Tokyo, Japan). Unless otherwise mentioned, all other chemicals were purchased from Sigma-Aldrich (St. Louis, MO, USA), Fujifilm Wako Pure Chemical Corporation (Osaka, Japan), Nacalai Tesque, Inc. (Kyoto, Japan), and Tokyo Chemical Industry Co., Ltd. (Tokyo, Japan).

### Plant materials

Seeds of onion cultivar ‘Sensyu’ were purchased from Noguchi Seed (Saitama, Japan). Seeds of onion cultivar ‘Getsurin’ and Welsh onion cultivar ‘Ishikura’ were purchased from Sakata Seed (Kanagawa, Japan). Seeds of Chinese chive cultivar ‘Hirohaba’ were purchased from Takii Seed (Kyoto, Japan).

Surface-sterilized seeds were germinated on half-strength Murashige and Skoog (MS) medium [22] containing 1% (w/v) sucrose and 0.2% (w/v) gellan gum, and grown at 20 °C under a 16-h light (750 lx.)/8-h dark photoperiod.

### Induction and culture of callus tissues

To initiate callus tissues, one-centimeter-length sections of leaves and roots of 3-week-old aseptic plants were cultured on MS medium containing 3% (w/v) sucrose and 0.2% (w/v) gellan gum, supplemented with picloram and 2-isopentenyladenine (2-iP) as auxin and cytokinin, respectively, at 25 °C under dark conditions. Nine different concentrations of picloram (1, 10, and 100 μM) and 2-iP (0, 1, and 10 μM) were tested. After 3 months, callus was induced from the plant sections. The induced callus tissues were maintained by sub-culturing every 2 months under the same conditions. Callus tissues stably maintained by multiple subcultures for more than 2 years were analyzed for their characteristics. The content of CSOs and alliinase activity was analyzed in callus tissues cultivated for 9 weeks after subculture. The before-hyphen part of the callus tissue line’s name indicates the organ (L, leaf; R, root) and the phytohormone condition used for callus induction.

### Extraction and analysis of CSOs

Extraction of CSOs from tissues was performed as previously described [12]. CSOs were quantitatively analyzed using LC–MS (LC, 1260 Infinity II; MS, 6470 LC/TQ; Agilent Technologies, Santa Clara, CA, USA) in the positive ion mode. LC was conducted using a Cadenza CD-C18 column (3.0 µm, 3.0 mm × 150 mm, Imtakt Corporation, Kyoto, Japan) following the previously described method [23]. (*N*-Morpholino)propanesulfonic acid was used as the internal standard.

### Alliinase activity assays

Tissues were homogenized in 50 mM sodium phosphate buffer (pH 7.0) containing 10% (w/v) glycerol at 4 °C using a mortar and pestle. The lysate was centrifuged at 10,000 × *g* for 5 min, and the supernatant was collected to measure alliinase activity. Protein concentration was determined using the Bio-Rad protein assay (Bio-Rad Laboratories, Inc., Hercules, CA, USA) based on a previously described method [24], with bovine serum albumin as the standard.

Alliinase activity was assayed by measuring pyruvate synthesized from methiin based on the standard method [25, 26] with some modifications as follows. For methiin, (±)-methiin (LKT Laboratories, St. Paul, MN, USA), which is a mixture of the natural form of methiin [(*R*_C_*S*_S_)]-*S*-methylcysteine sulfoxide] and its diastereomer [(*R*_C_*R*_S_)]-*S*-methylcysteine sulfoxide] was used. The standard enzyme reaction mixture consisted of 1 µg µl^−1^ plant crude protein, 50 mM sodium phosphate (pH 7.0), and 10 mM (±)-methiin in a final volume of 100 µl was incubated for 2–29 min, according to the level of alliinase activity of each sample, at 25 °C. The enzymatic reaction was terminated by adding 100 µl of 10% (w/v) trichloroacetic acid to the reaction mixture. After the denatured protein was removed by centrifugation, 180 µl of supernatant was collected and mixed with 180 µl of 0.025% (w/v) 2,4-dinitrophenylhydrazine dissolved in 1 M HCl. The solution was incubated for 5 min at 25 °C, and 180 µl of 1.5 M NaOH was added. The absorbance at 520 nm was determined using a UV/VIS spectrophotometer U-2001 (Hitachi High-Tech Corp., Tokyo, Japan). Pyruvate concentration was calculated using the standard curve of pyruvate.

## Results and discussion

### Establishment of callus tissue cultures from *Allium* plants

Callus tissues were newly induced from three *Allium* plants: onion, Welsh onion, and Chinese chive. For the onion, an autumn-sown cultivar ‘Sensyu’ suitable for cultivation in warm regions of Japan and a spring-sown cultivar ‘Getsurin’ suitable for cultivation in cold regions of Japan were used. For callus induction, the leaf and root segments of 3-week-old plants were placed on a medium containing picloram and 2-iP as auxin and cytokinin, respectively. Nine phytohormone conditions, Pi-1 to Pi-9, which have different concentrations of picloram (1, 10, and 100 μM) and 2-iP (0, 1, and 10 μM), were tested (Table 1). After 3 months of culture, callus tissues emerged from all the plant species tested. Callus tissues were maintained in multiple subcultures.

**Table 1:** The number of established lines of callus induced from onion, Welsh onion, and Chinese chive

Condition name	Phytohormone concentration		Number of established callus lines							
	Picloram (µM)	2-iP (µM)	Onion cultivar ‘Sensyu’		Onion cultivar ‘Getsurin’		Welsh onion		Chinese chive	
			Leaf	Root	Leaf	Root	Leaf	Root	Leaf	Root
Pi-1	1	0	0	3	0	5	0	1	0	0
Pi-2	10	0	0	4	2	2	0	7	0	0
Pi-3	100	0	0	0	1	0	1	5	0	0
Pi-4	1	1	0	2	0	1	0	0	0	0
Pi-5	10	1	0	6	1	2	0	5	1	2
Pi-6	100	1	0	1	0	0	2	4	0	0
Pi-7	1	10	0	3	0	0	0	0	0	2
Pi-8	10	10	0	1	0	1	0	2	0	4
Pi-9	100	10	0	0	0	0	0	1	1	3

**Fig. 2:**
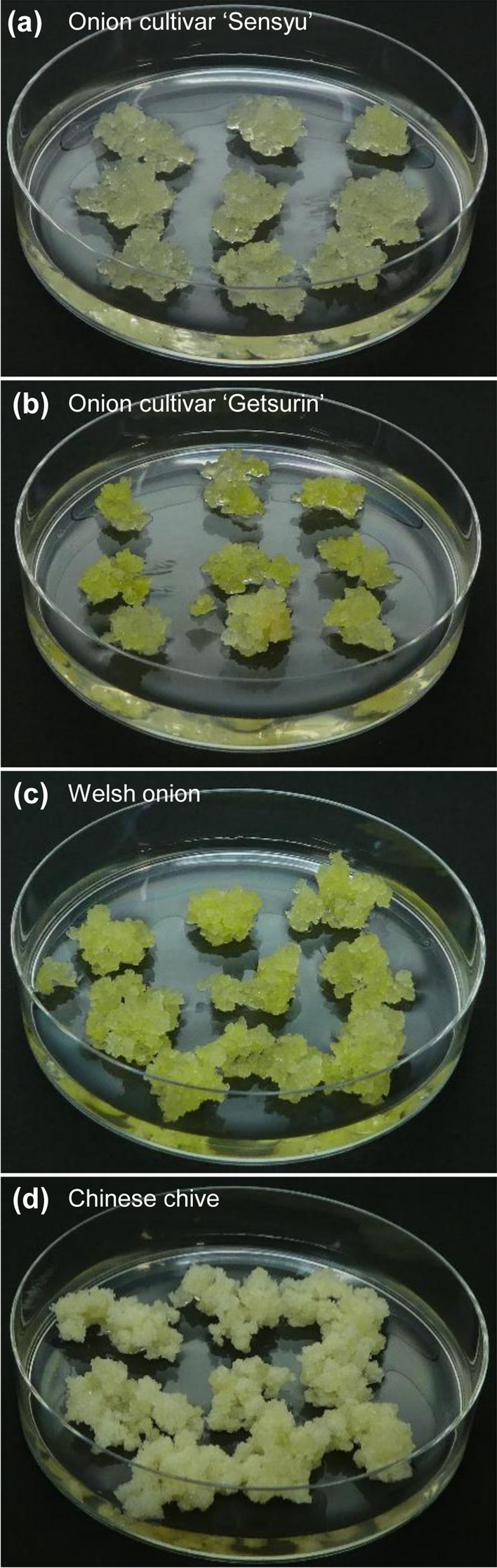
The representative callus tissues established in this study. Callus tissues cultivated for 4 weeks after subculture are shown. **a** Onion cultivar ‘Sensyu’, line R5-1. **b** Onion cultivar ‘Getsurin’, line R2-2. **c** Welsh onion, line R5-5. **d** Chinese chive, line R8-B2

Generally, the callus tissues of onion were yellowish-white with relatively fine cells, regardless of cultivar (Fig. 2a, b). The callus tissues of Welsh onion were yellow and formed hard cell clumps (Fig. 2c). The callus tissues of Chinese chive were white with soft cotton-like cells intimately intertwined (Fig. 2d) and tended to be re-differentiated to form adventitious roots.

The number of callus tissue lines stably maintained for more than 2 years is summarized in Table 1. Among the nine phytohormone conditions tested, high-quality callus tissues showing high growth rates were efficiently obtained under the conditions of Pi-2, Pi-5, and Pi-8, suggesting that the presence of 10 μM picloram in the medium was suitable for the induction and growth of callus tissues (Table 1). High-quality callus tissues were efficiently induced also under the condition of Pi-6. In contrast, callus tissues established under the conditions of Pi-1, Pi-4, and Pi-7 showed relatively poor growth. For all plants tested, callus tissues were more efficiently induced from the roots than from the leaves, irrespective of phytohormone conditions, except for those from onion cultivar ‘Getsurin’ under the conditions of Pi-2 and Pi-3 (Table 1). For each plant, approximately 10 callus tissue lines showing high growth rates were selected to analyze their CSOs levels and alliinase activities.

## Quantification of CSOs

**Fig. 3:**
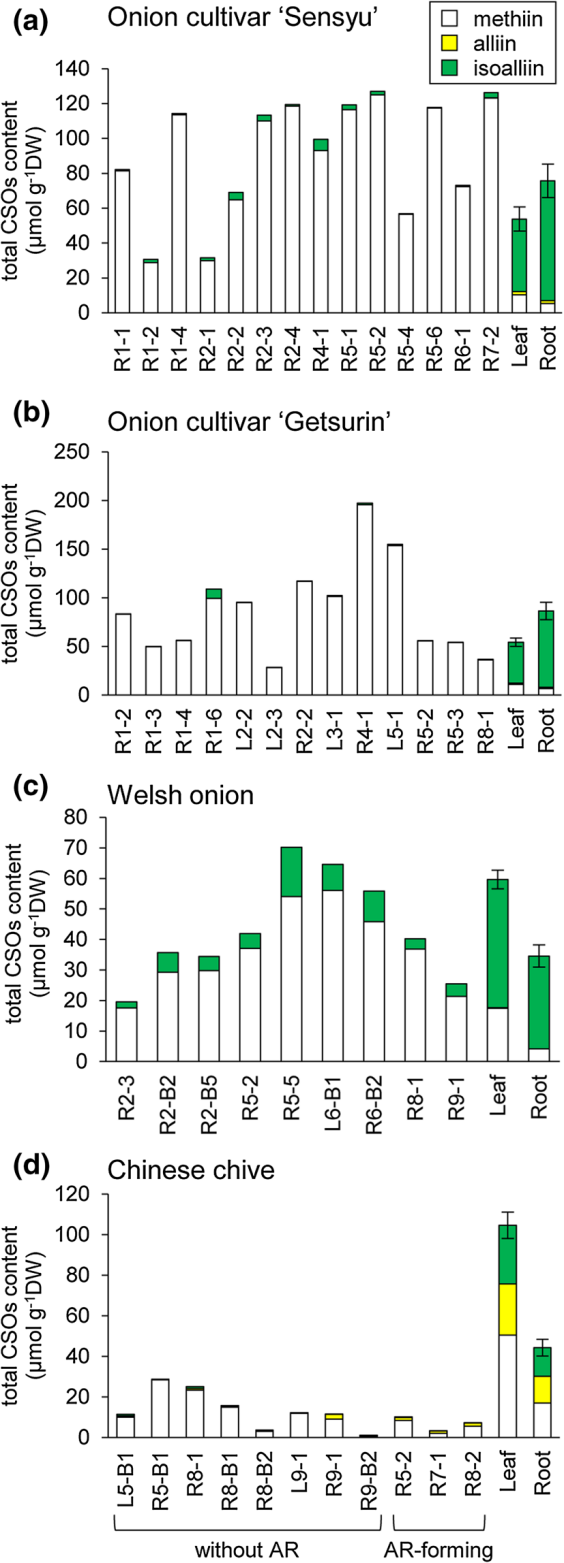
The levels of CSOs in the callus tissues and the intact plants. The content of methiin, alliin, and isoalliin in the callus tissues cultivated for 9 weeks after subculture, and the leaves and roots of 5-week-old onion cultivar ‘Sensyu’ **(a)** and onion cultivar ‘Getsurin’ **(b)**, Welsh onion **(c)**, and Chinese chive **(d)** was measured using LC–MS. The before-hyphen part of the callus tissue line’s name indicates the organ (L, leaf; R, root) and the phytohormone condition used for callus induction. For example, R1 indicates callus induced from roots under the condition of Pi-1. Among 11 callus tissues of Chinese chive tested, three callus tissues formed adventitious roots (AR). Leaf and root data from the intact plants represent the mean ± SD of five biological replicates

The content of CSOs in the callus tissues cultivated for 9 weeks after subculture was measured using LC–MS and compared with that in the leaves and roots of 5-week-old intact plants (Fig. 3). In the intact onion plants, a large amount of isoalliin, a smaller amount of methiin, and a trace amount of alliin were detected in leaves and roots, regardless of cultivar (Fig. 3a, b). In the intact Welsh onion plants, isoalliin was a major CSO, while methiin was a minor CSO in leaves and roots (Fig. 3c). Chinese chive contained similar levels of methiin, alliin, and isoalliin in the leaves and roots (Fig. 3d). These results are consistent with previously reported findings [27–30].

In contrast to the substantial accumulation of isoalliin in the intact plants, the major CSOs in the callus tissues were methiin in all plant species tested (Fig. 3). Although the content of total CSOs ranged widely among callus tissue lines, the content of methiin was generally higher in the callus tissues than in the intact plants for onion and Welsh onion (Fig. 3a–c). Methiin levels in the callus tissues were similar or lower than the intact plants for Chinese chive (Fig. 3d). The ratio of methiin levels of the callus tissues to that of the intact plant leaves was 278–1207% in onion cultivar ‘Sensyu’, 254–1758% in onion cultivar ‘Getsurin’, 100–321% in Welsh onion, and 0–56% in Chinese chive. In contrast, the content of alliin and isoalliin in the callus tissues was much lower than that in the intact plants, regardless of plant species (Fig. 3). The total CSOs levels in the callus tissues were generally comparable with those in the intact plants for onion and Welsh onion (Fig. 3a–c), while the levels were lower in the callus tissues than in the intact plants for Chinese chive (Fig. 3d). The total CSOs levels of the callus tissues to that of leaves from the intact plants were 57–236% in onion cultivar ‘Sensyu’, 52–363% in onion cultivar ‘Getsurin’, 33–118% in Welsh onion, and 1–27% in Chinese chive.

Our results showing that the callus tissues accumulated a substantial amount of methiin and low amounts of alliin and isoalliin are largely consistent with previous reports that analyzed a single representative line of undifferentiated callus tissue by TLC for garlic [19] and onion [18]. Although the amount of methiin in the callus tissues was similar to or lower than that in the intact plants in these previous studies [18, 19], most of the callus lines of onion and Welsh onion established in the present study showed much higher content of methiin compared to the intact plants (Fig. 3a–c). This difference among the studies likely reflects the differences in the species and cultivar used to induce the callus tissues, the method for inducing and maintaining the callus tissues, and the number of callus tissue lines analyzed. Our results indicate that the cellular developmental conditions and probably the regulatory mechanism necessary for the biosynthesis of methiin are different from alliin and isoalliin. This idea is consistent with the proposed biosynthetic pathway that the methiin biosynthesis separates from the routes for alliin and isoalliin at the initial stage (Fig. 1). The cellular concentration of methiin is estimated to be 3.2–13.9 mM in the callus tissues of onion cultivar ‘Sensyu’, 3.1–21.7 mM in the callus tissues of onion cultivar ‘Getsurin’, 1.9–6.2 mM in the callus tissues of Welsh onion, and 0.03–3.1 mM in the callus tissues of Chinese chive, when the water content of the tissues is deduced to be 90%. This finding is surprising since the concentration of methiin in the callus tissues was considered to be comparable to that of glutathione (0.1–10 mM), a major non-protein sulfur-containing compound, in typical cells [31]. The ability of callus tissues to produce a large amount of methiin may imply the role of methiin not only as a defense compound against natural enemies, such as bacteria and fungi, but also as a storage form of sulfur for *Allium* plants, as in the case of glucosinolates for Brassicaceae species [32].

### Alliinase activity analysis

**Fig. 4:**
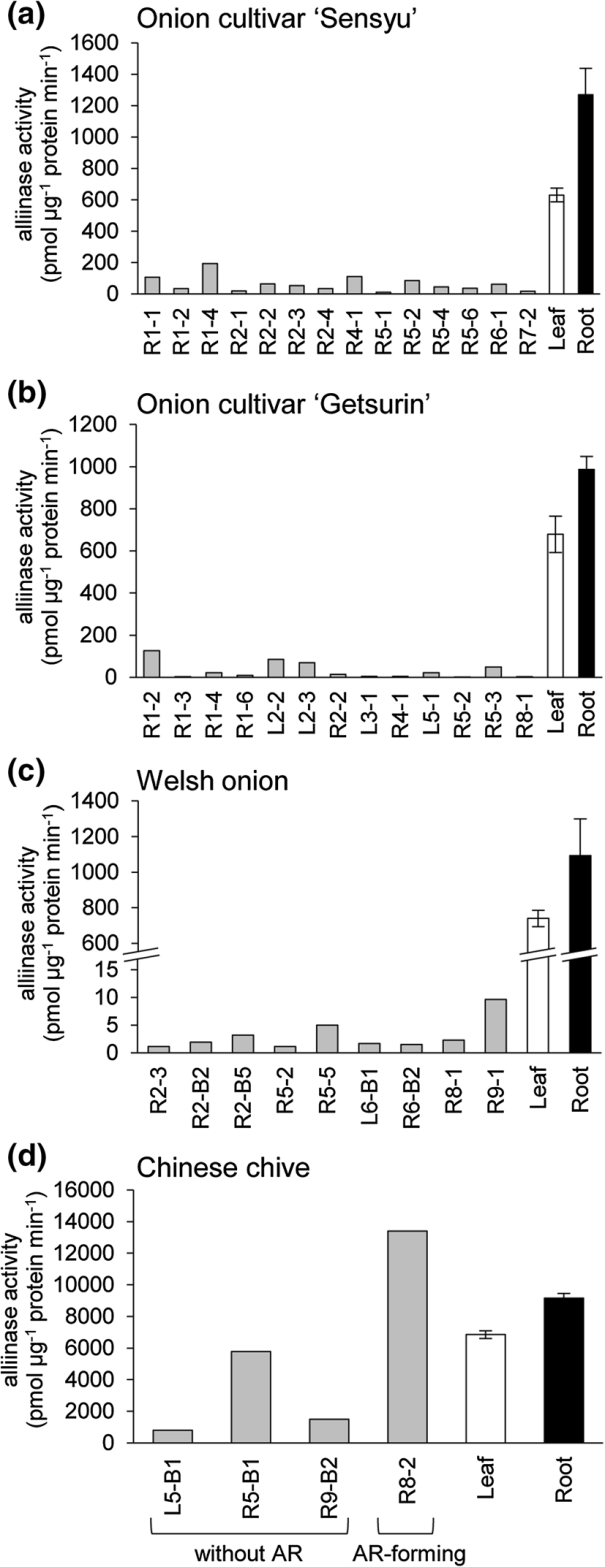
Alliinase activity in the callus tissues and the intact plants. Alliinase activity was measured for the protein extracted from the callus tissues cultivated for 9 weeks after subculture, and the leaves and roots of 5-week-old onion cultivar ‘Sensyu’ **(a)** and onion cultivar ‘Getsurin’ **(b)**, Welsh onion **(c)**, and Chinese chive **(d)**. Leaf and root data from the intact plants represent the mean ± SD of five biological replicates

Protein extracts were prepared from the callus tissues cultivated for 9 weeks after subculture and 5-week-old intact plants and assayed for alliinase activity against methiin (Fig. 4). Since protein quantification was unsuccessful for 7 of the 11 tested callus tissues of Chinese chive, four callus tissues, including one adventitious root-forming callus tissue, were analyzed.

Alliinase activity was detected in the leaves and roots of all the plant species analyzed (Fig. 4). Chinese chives showed approximately 10-fold higher alliinase activity than onion and Welsh onion. In onion callus tissues, alliinase activity was less than 30% of that in leaves, regardless of cultivar (Fig. 4a, b). Welsh onion callus tissues showed almost negligible alliinase activity (Fig. 4c). In contrast, alliinase activity in Chinese chive callus tissues ranged widely. The alliinase activity of the callus tissues L5-B1, R5-B1, and R9-B2 was 11–85% of the leaf levels, while that of the adventitious root-forming callus tissue R8-2 was 196% of the leaf levels (Fig. 4d).

Previous studies have revealed that alliinase shows tissue- and organelle-specific localization in *Allium* plants. Alliinase is almost exclusively localized in the vascular bundle sheath cells at the tissue level, at least in onion, Chinese chive, and garlic [6, 7]. In cells, alliinase is sequestered in the vacuole [5]. Electron microscopy revealed that cells and central vacuoles in onion bulb tissues, which showed high alliinase activity, were larger than those in onion callus tissues [18]. The development of these structures is considered to be essential for the expression and accumulation of alliinases. It is most likely that the low alliinase activity observed for the callus tissues of onion and Welsh onion (Fig. 4a–c) was attributed to the insufficient accumulation of alliinase in these undifferentiated cells. In contrast, the callus tissues of Chinese chive showed alliinase activity at levels similar to those of the intact plants (Fig. 4d). Since only the callus tissues of Chinese chive developed adventitious roots in our study, they might tend to be re-differentiated compared to those of onion and Welsh onion.

Our data demonstrated that the callus tissues of onion and Welsh onion accumulated high levels of methiin and showed low alliinase activity compared to their corresponding intact plants (Figs. 3 and 4). Deficiency in alliinase activity in these callus tissues could minimize the enzymatic degradation of methiin during its extraction from cells for industrial purposes. In addition to generating bioactive compounds through alliinase-mediated degradation, methiin itself is valuable as a natural product with antioxidative and anti-hypercholesterolemic effects [33, 34]. The present study revealed the potential of utilizing the callus tissues of onion and Welsh onion as an efficient production system for methiin.

## Conclusion

In this study, callus tissues of onion, Welsh onion, and Chinese chive were successfully established and analyzed for their CSOs levels and alliinase activities. Among the nine phytohormone conditions tested, Pi-2, Pi-5, Pi-6, and Pi-8, three of which contained 10 μM picloram as auxin, were efficient in inducing high-quality callus tissues. Unlike intact plants, the callus tissues almost exclusively contained methiin as CSO, irrespective of plant species, implying that the regulatory mechanism for the biosynthesis of methiin is different from that of alliin and isoalliin. Moreover, because of the high methiin content and low alliinase activity, the callus tissues of onion and Welsh onion could be promising for application as a plant-based system for methiin production.
