# Correction to: The ability of callus tissues induced from three *Allium* plants to accumulate health-beneficial natural products, *S*-alk(en)ylcysteine sulfoxides

**DOI:** 10.1007/s11418-023-01692-z

**Published:** 2023-03-15

**Authors:** Naoko Yoshimoto, Takashi Asano, Ayuna Kisanuki, Chihiro Kanno, Machiko Asanuma, Mami Yamazaki, Isao Fujii, Kazuki Saito

**Affiliations:** 1 Graduate School of Pharmaceutical Sciences, Chiba University, 1-8-1 Inohana, Chuo-ku, Chiba, 260-8675 Japan; 2 Plant Molecular Science Center, Chiba University, 1-8-1 Inohana, Chuo-ku, Chiba, 260-8675 Japan; 3 School of Pharmacy, Iwate Medical University, 1-1-1 Idaidori, Yahaba, Iwate, 028-3694 Japan; 4 RIKEN Center for Sustainable Resource Science, 1-7-22 Suehiro-cho, Tsurumi-ku, Yokohama, 230-0045 Japan

**Correction to: Journal of Natural Medicines (2022) 76:803–810** 10.1007/s11418-022-01631-4

The article The ability of callus tissues induced from three *Allium* plants to accumulate health-beneficial natural products, *S*-alk(en)ylcysteine sulfoxides, written by Naoko Yoshimoto, Takashi Asano, Ayuna Kisanuki, Chihiro Kanno, Machiko Asanuma, Mami Yamazaki, Isao Fujii, Kazuki Saito, was originally published Online First without Open Access. After publication in volume 76, issue 4, page 803–810 the author decided to opt for Open Choice and to make the article an Open Access publication. Therefore, the copyright of the article has been changed to © The Author(s) 2023 and the article is forthwith distributed under the terms of the Creative Commons Attribution 4.0 International License, which permits use, sharing, adaptation, distribution and reproduction in any medium or format, as long as you give appropriate credit to the original author(s) and the source, provide a link to the Creative Commons licence, and indicate if changes were made. The images or other third party material in this article are included in the article’s Creative Commons licence, unless indicated otherwise in a credit line to the material. If material is not included in the article’s Creative Commons licence and your intended use is not permitted by statutory regulation or exceeds the permitted use, you will need to obtain permission directly from the copyright holder. To view a copy of this licence, visit http://creativecommons.org/licenses/by/4.0/.

The original article has been updated.

